# Self-Immolation in the Arab World: A Systematic Review

**DOI:** 10.1192/j.eurpsy.2022.2168

**Published:** 2022-09-01

**Authors:** S. El Hayek, M. Cherro, N. El Harake, E. Ghossoub

**Affiliations:** 1 University of Miami Miller School of Medicine, Psychiatry And Behavioral Sciences, Miami, United States of America; 2 American University of Beirut, Psychiatry, Beirut, Lebanon

**Keywords:** Suicide, self-immolation, arab world

## Abstract

**Introduction:**

Self-immolation is the centuries-old act of setting fire to oneself. Recent spikes in self-immolation events have been noticed in the Arab world, specifically in the aftermath of the Arab Spring in 2011.

**Objectives:**

To examine the literature assessing the characteristics and patterns of suicide by self-immolation in the Arab world.

**Methods:**

We registered our systematic review in Prospero [CRD42020207164]. We searched PubMed, Medline, PsycInfo, Embase, and Scopus databases from inception until 6 September 2021. We collected relevant articles via title and abstract screening followed by full-text screening. We then conducted a narrative synthesis of the results.

**Results:**

We found 31 out of 314 articles that fit our inclusion criteria: 4 qualitative and 27 quantitative cross-sectional studies. The quantitative studies had a sample size ranging from 22 to 600 self-inflicted burn victims. The studies emanated from Iraq (n=16), Tunisia (n=6), Saudi Arabia (n=2), Libya (n=2), Jordan (n=2), and Egypt, Palestine and Bahrain collectively (n=3). Studies showed that self-immolators are commonly married females, age ranging between 13 and 66 years old, having no education or solely primary education, and of low socioeconomic status. Self-immolation was more likely to happen at home, usually following marital conflicts, with the primary motive being suicide. Studies highlighted an increase of self-immolation as a form of protest. Kerosene was the most commonly used accelerant. Depression was the most comorbid mental health diagnosis.

**Conclusions:**

Self-immolation is not uncommon in the Arab world. Specific interventions directed at the population at risk are warranted.

**Disclosure:**

No significant relationships.

